# Author Correction: Spatial proteomics of ovarian cancer precursors delineates early disease changes and drug targets

**DOI:** 10.1038/s44320-025-00178-2

**Published:** 2025-12-17

**Authors:** Anuar Makhmut, Mihnea P Dragomir, Sonja Fritzsche, Markus Moebs, Wolfgang D Schmitt, Eliane T Taube, Fabian Coscia

**Affiliations:** 1 Max-Delbrück-Center for Molecular Medicine in the Helmholtz Association (MDC), Spatial Proteomics Group, Berlin, Germany; 2 Charité – Universitätsmedizin Berlin, corporate member of Freie Universität Berlin and Humboldt- Universität zu Berlin, Berlin, Germany; 3 Charité – Universitätsmedizin Berlin, corporate member of Freie Universität Berlin and Humboldt-Universität zu Berlin, Institute of Pathology, Berlin, Germany; 4 Berlin Institute of Health at Charité – Universitätsmedizin Berlin, Berlin, Germany; 5 German Cancer Consortium (DKTK), Partner Site Berlin, German Cancer Research Center (DKFZ), Heidelberg, Germany; 6 Humboldt-Universität zu Berlin, Institute of Biology, Berlin, Germany; 7 Present Address: Institute of Pathology, Diagnostik Ernst von Bermann, Potsdam, Germany

## Abstract

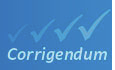

**Correction to:** *Molecular Systems Biology* (2025). 10.1038/s44320-025-00168-4 | Published online 13 November 2025

**The Data Availability section is corrected**.

**Dataset EV9 legend is corrected**.

The data availability section is corrected to reflect the correct dataset identifier.

The correct dataset identifier is corrected from PXD069360.

To: PXD069630.

Dataset EV9 is corrected from:

Student’s t-test results used for differential abundance analysis of PAX8-positive NFTE and PAX8-negative NFTE samples and visualized in the volcano plot figure EV3F.

To: (Changes in bold)

Student’s t-test results used for differential abundance analysis **of STICs and PAX8-positive** NFTE samples and visualized in the volcano plot figure **EV3G**.

The HTML has been updated to include a corrected version of the Dataset EV9.

## Supplementary information

Updated Dataset EV9

