# Supplementary material for: Spatial proteomics of ovarian cancer precursors delineates early disease changes and drug targets
Source: Mol Syst Biol. 2025 Nov 13;22(1):7–41. doi: 10.1038/s44320-025-00168-4 (PMC12759074; doi:10.1038/s44320-025-00168-4)
Supplement: Supplementary file 1 — Appendix [file 44320_2025_168_MOESM1_ESM.pdf]

Appendix for

Spatial proteomics of ovarian cancer precursors delineates early disease changes and drug targets

by Makhmut & Dragomir et al.

Table of Contents

Appendix Figures

Appendix figure S1 – Comparison of overall survival between cluster 1 tumor patients (turquoise) and cluster 2 tumor patients (dark blue)……………………………………………………………………………**Page 1**

Appendix figure S2 – Pairwise comparison between cluster 1 (turquoise) and cluster 2 (dark blue) STIC/tumor stroma samples………………………………………………………………………………………..**Page 2**

Appendix figure S3 – Heatmap of average gene expression levels of eight FDA-approved drug targets across seven cell types……………………………………………………………………………………………………..**Page 3**

Appendix figure S4 – Example of image segmentation for patients W31V and W30FW30D invasive carcinoma regions……..**Page 4**

Appendix figure S5 – Quantification of marker-positive cells in histological tissue section………..**Page 5**

Appendix Tables

Appendix table S1 - Synergy scores for all tested drug combinations……………………………………….**Page 6**

Appendix table S2 - Exact p-values of Fig. 2I…………………………………………………………………………**Page 6**

Appendix table S3 - Exact p-values of Fig. 6C…………………………………………………………………………**Page 6**

Appendix table S4 - Exact p-values of Fig. 6G…………………………………………………………………………**Page 7**

Appendix table S5 - Exact p-values of Fig. 7B…………………………………………………………………………**Page 7**

Appendix Methods

Cell segmentation and marker quantification, related to Fig 4F-G…………………………………………….**Page 7**

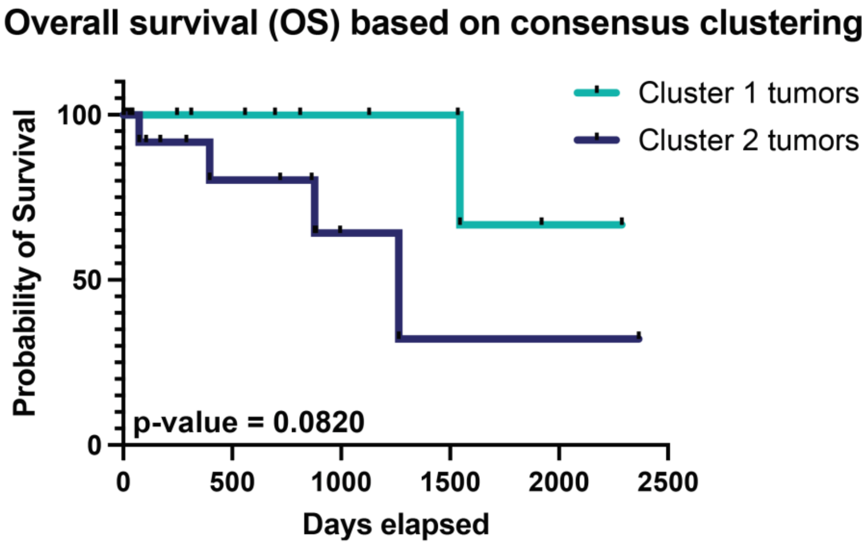

Appendix figure S1: *Kaplan Meier plot comparing overall survival between patients with cluster 1 tumors (turquoise) and cluster 2 tumors (dark blue). The x-axis indicates time in days, and the y-axis shows the estimated probability of survival. Significance levels were determined using the log-rank test.*

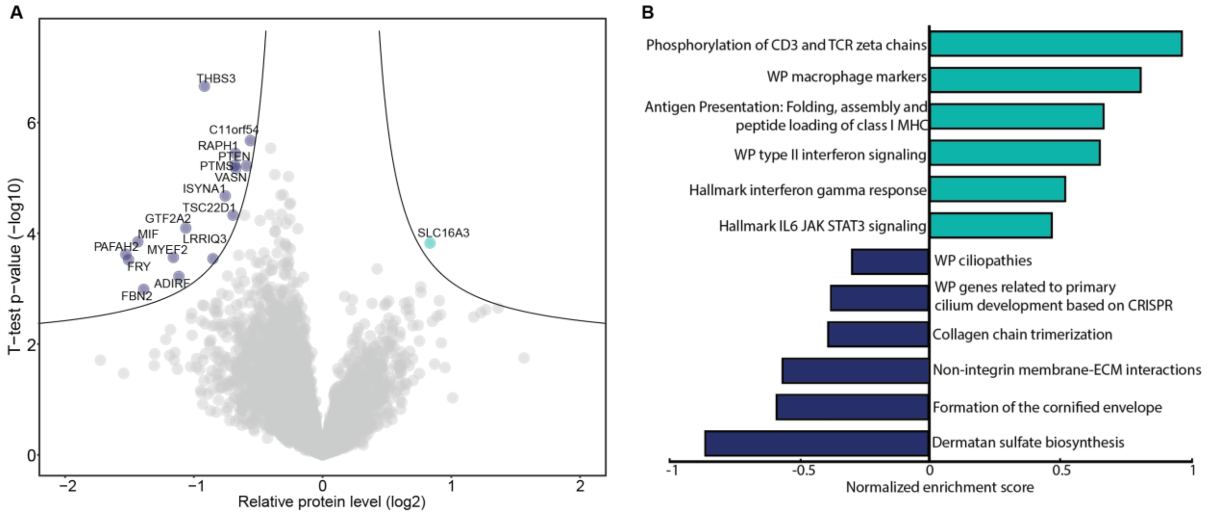

Appendix figure S2: *A) Volcano plot of pairwise comparison between cluster 1 (turquoise) and cluster 2 (dark blue) STIC/tumor stroma samples. Proteins with the highest fold changes are highlighted (two-sided Student's t-test, false discovery rate [FDR] < 0.05). B) Pathway enrichment analysis (WikiPathways, Hallmarks, Reactome) based on the t-test difference between cluster 1 and cluster 2 epithelial samples. Selected pathways with a Benjamin-Hochberg FDR < 0.05 are shown.*

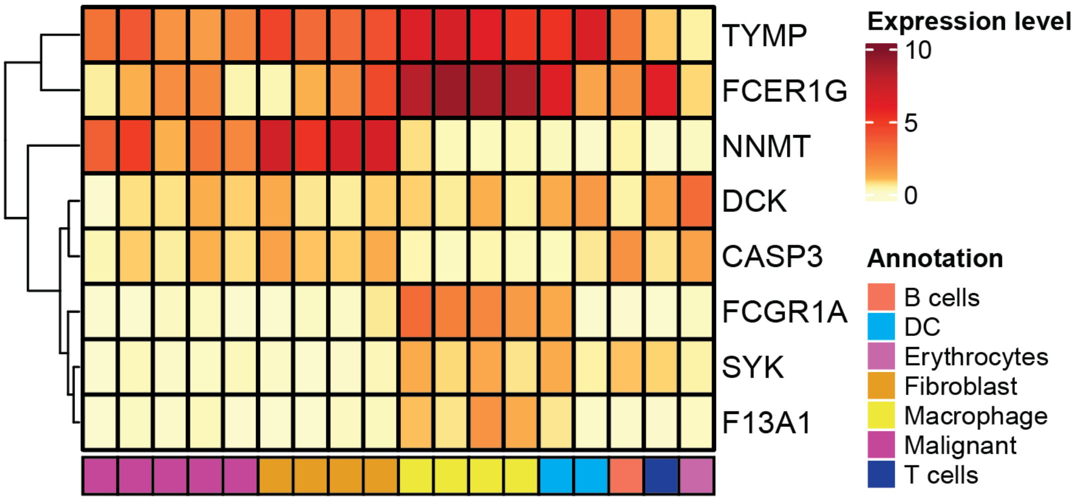

Appendix figure S3: *Heatmap of average gene expression levels of eight FDA-approved drug targets across seven cell types. Rows represent genes, and columns represent distinct clusters annotated by cell type. Expression levels are indicated by color intensity (red – higher expression, yellow – lower expression). Data was obtained from Izar et al. (PMID: 32572264).*

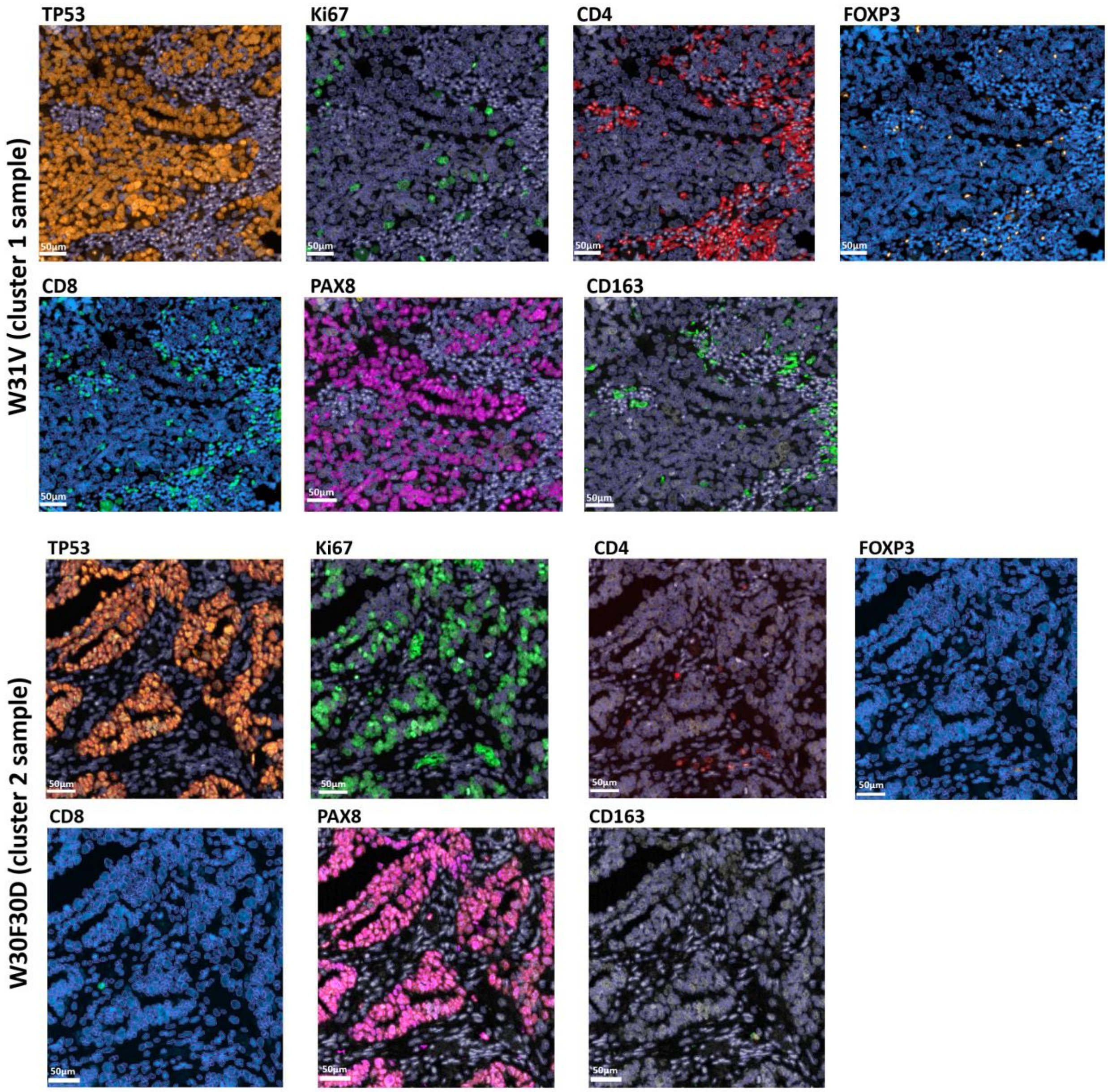

Appendix figure S4: *Representative immunofluorescence microscopy image of cancer tissue sections from patients W31V (cluster 1, IMR subtype, upper panel) and W30W30D (cluster 2, PRO subtype, lower panel), showing TP53, Ki67, CD4, FOXP3, CD8, PAX8, CD163 and DAPI. Cell segmentation was performed using QuPath software to identify individual cells within the tissue. Segmented cells were subsequently analyzed to determine the proportion of marker-positive cells relative to the total number of cells. Scale bar: 50 µm.*

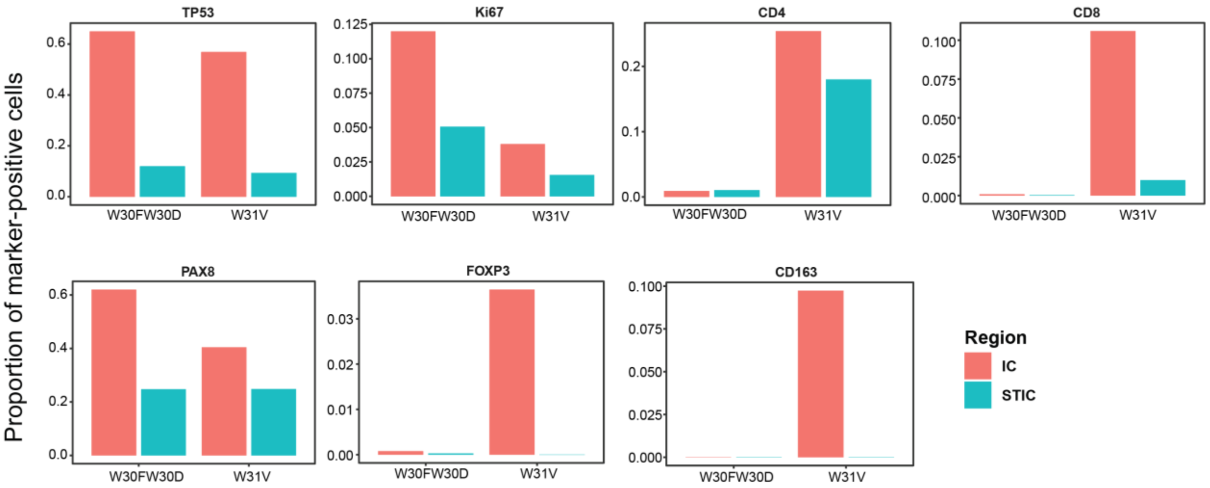

Appendix figure S5: *Quantification of marker-positive cells in histological tissue sections from patients W31V (cluster 1, IMR subtype) and W30W30D (cluster 2, PRO subtype). Immunofluorescence staining was performed to detect various markers across tissue sections (as in Figure 4F-H). The proportion of marker-positive cells was quantified for each marker using Qupath. Each value represents the ratio of cells positive for a given marker to the total number of DAPI-positive nuclei. Markers analyzed include TP53, Ki67, CD4, CD8, PAX8, FOXP3, CD163. Boxplot represent the proportion of marker-positive cells for each marker. Data represent a single experimental replicate per sample.*

Cell line	Drug combination	HSA Synergy Score (Mean)<br>*** p-value < 0.001
**ES-2**	Carboplatin - AY9944	7.52 ***
**EFO-21**	Carboplatin - AY9944	13.5 ***
**OVCAR-8**	Carboplatin - AY9944	14.9 ***
**OAW-42**	Carboplatin - AY9944	2.38 n.s.
**ES-2**	Carboplatin - Simvastatin	7.9 ***
**EFO-21**	Carboplatin - Simvastatin	9.36 ***
**OVCAR-8**	Carboplatin - Simvastatin	-3.52 ***
**OAW-42**	Carboplatin - Simvastatin	4.64 ***

***Appendix table S1**: Synergy scores for all tested drug combinations. An HSA score of greater than 10 indicates high synergy.*

Protein/Marker	NFTE – STIC	STIC – IC	NFTE – IC
MKI67	3.1×10^{-7} (***)	0.68 (ns)	3.8×10^{-6} (***)
TP53	5.2×10^{-5} (***)	0.98 (ns)	1.6×10^{-6} (***)
NNMT (stroma)	0.0088 (**)	4.4×10^{-5} (***)	2.4×10^{-8} (***)
CD163 (stroma)	0.00015 (***)	0.0037 (**)	1.5×10^{-9} (***)
VCAN (stroma)	0.001 (**)	5.37×10^{-7} (***)	0.001 (**)

***Appendix table S2**: Related to Fig. 2I. Exact p-values.*

Pathway	NFTE – STIC	STIC – IC	NFTE – IC
Prostaglandin biosynthesis	$p < 2.2 \times 10^{-16}$ (***)	0.054 (ns)	$p < 2.2 \times 10^{-16}$ (***)
OxPhos	$p < 2.2 \times 10^{-16}$ (***)	0.0014 (**)	$p < 2.2 \times 10^{-16}$ (***)
Glycolysis & Gluconeogenesis	0.63 (ns)	7.8×10^{-14} (***)	6.1×10^{-14} (***)
Ribosomal proteins	0.963 (ns)	$p < 2.2 \times 10^{-16}$ (***)	$p < 2.2 \times 10^{-16}$ (***)
Type I collagen synthesis	1×10^{-8} (***)	0.00071 (***)	4×10^{-15} (***)
Cholesterol biosynthesis	2.3×10^{-15} (***)	0.64 (ns)	2.7×10^{-15} (***)
DNA replication	$p < 2.2 \times 10^{-16}$ (***)	0.007 (**)	$p < 2.2 \times 10^{-16}$ (***)
DNA mismatch repair	0.00074 (***)	0.029 (*)	1.5×10^{-7} (***)

***Appendix table S3**: Related to Fig.6C. Exact p-values.*

Protein	NFTE – STIC	STIC – IC	NFTE – IC
DHCR24	p < 0.0001 (***)	0.4482 (ns)	p < 0.0001 (***)
DHCR7	p < 0.0001 (***)	0.9261 (ns)	p < 0.0001 (***)

***Appendix table S4**: Related to Fig.6G. Exact p-values.*

Comparison	OVCAR8	EFO21	ES2	OAW-42
Vehicle vs Simvastatin	<0.0001 (****)	0.0274 (*)	0.0015 (**)	0.1036 (ns)
Vehicle vs AY9944	0.0038 (**)	0.0033 (**)	<0.0001 (****)	0.0137 (*)
Vehicle vs Carbo 1 µM	0.5206 (ns)	0.3092 (ns)	0.0185 (*)	0.02695 (*)
Vehicle vs Carbo 10 µM	0.1936 (ns)	0.1356 (ns)	0.1025 (ns)	0.5783 (ns)
Vehicle vs Carbo 100 µM	0.0476 (*)	0.0046 (**)	0.0041 (**)	0.0013 (**)
Vehicle vs Carbo 150 µM	0.0259 (*)	<0.0001 (****)	0.0009 (***)	<0.0001 (****)

***Appendix table S5**: Related to Fig.7B. Exact p-values.*

Appendix methods

Cell segmentation and marker quantification

Cell detection and classification were performed using QuPath image analysis software. First, the *Cell Detection* function was applied to identify nuclei within the tissue section, using the DAPI channel as the detection channel. Default nucleus parameters were applied, while intensity thresholds were adjusted between 75–100 depending on the quality of DAPI staining. The cell expansion parameter was set to 2 µm to approximate whole-cell boundaries, and all other general parameters were kept at default settings.

For marker classification, a Single Measurement Classifier was trained for each channel of interest (e.g., TP53, PAX8, CD8, CD4, FOXP3, Ki67, CD163). The Object filter was set to "Detections (all)", and the Channel filter was applied to the corresponding fluorescence channel. Depending on the subcellular localization of the antibody, the measurement parameter was defined as either nucleus (mean intensity) or cytoplasm (mean intensity). Classification thresholds were optimized individually for each marker to reliably distinguish marker-positive from marker-negative cells.
