# Supplementary material for: Spatial proteomics of ovarian cancer precursors delineates early disease changes and drug targets
Source: Mol Syst Biol. 2025 Nov 13;22(1):7–41. doi: 10.1038/s44320-025-00168-4 (PMC12759074; doi:10.1038/s44320-025-00168-4)
Supplement: Supplementary file 28 — Expanded View Figures [file 44320_2025_168_MOESM28_ESM.pdf]

Expanded View Figures

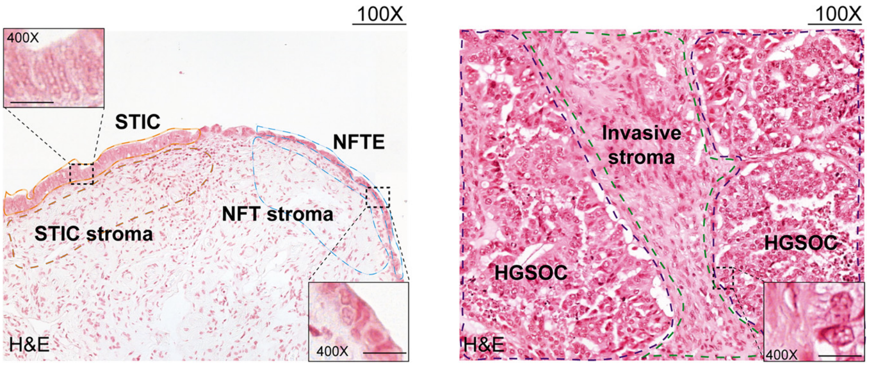

Figure EV1. Pathology-guided ultra-low input proteomics of HGSOC precursor lesions.

Representative H&E stains of normal fallopian tube epithelium (NFTE), serous tubal intraepithelial carcinoma (STIC), and invasive carcinoma (IC) and corresponding epithelial and stromal compartments. Scale bars: 100X: 100 µm, 400X: 20 µm.

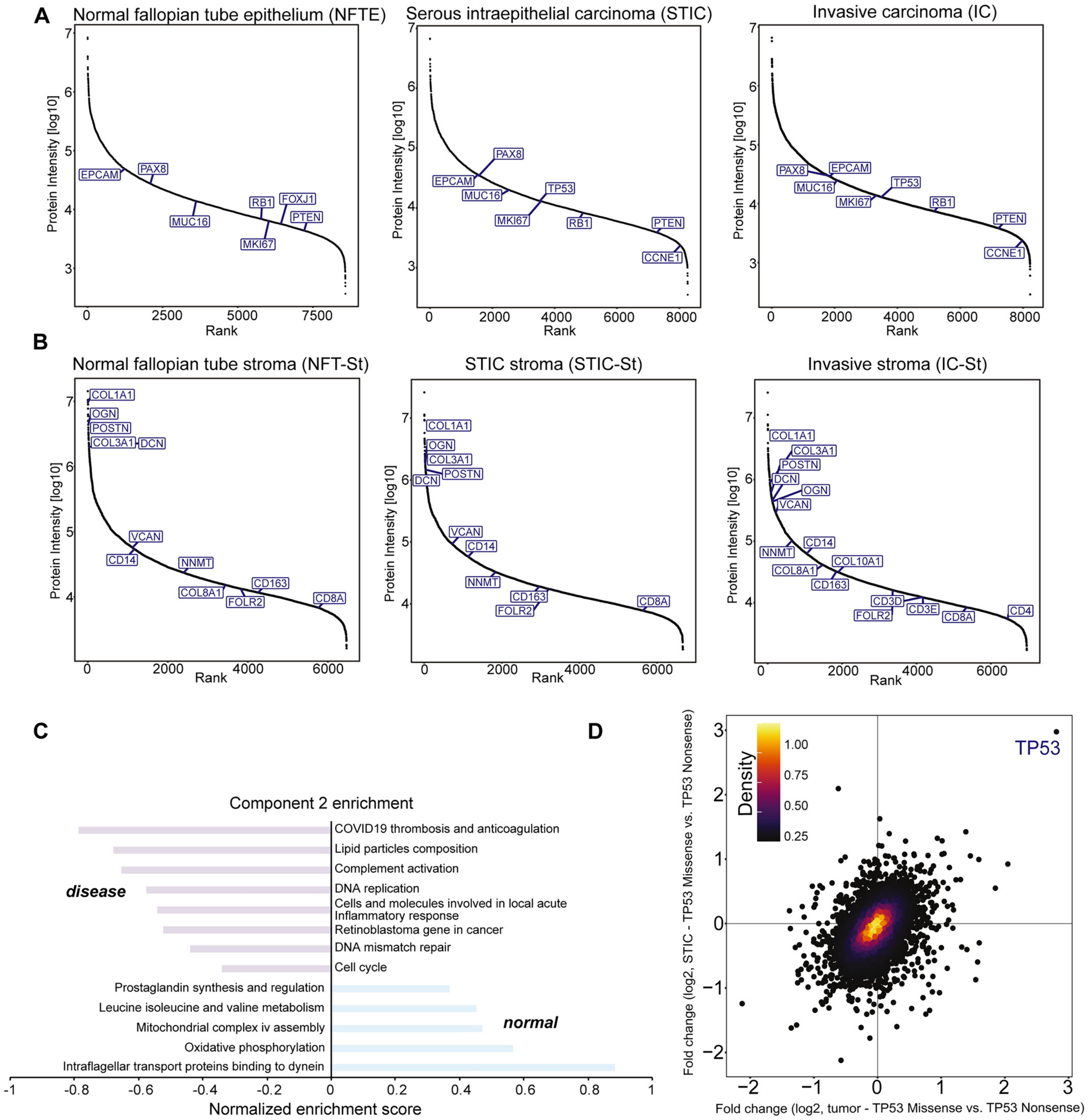

Figure EV2. Spatially resolved proteomes reflect disease-specific alterations at the bulk level depth.

(**A**, **B**) Dynamic range of median protein abundance for epithelial (**A**) and stromal (**B**) compartment samples. Known ovarian cancer-, cell type-, and stromal markers are highlighted. A minimum of 18 quantified values were required for each marker to be displayed. Proteins with 50% valid values for each group are shown. (**C**) Pathway enrichment analysis (Hallmarks) based on PC2 reveals differences between normal and disease compartments. Selected terms with a Benjamini–Hochberg FDR <0.05 are shown. (**D**) Scatter plot of *t*-test results between p53 overexpression and p53 null mutation patients, indicating that p53 is the only differentially abundant protein between the two groups.

 © The Author(s)

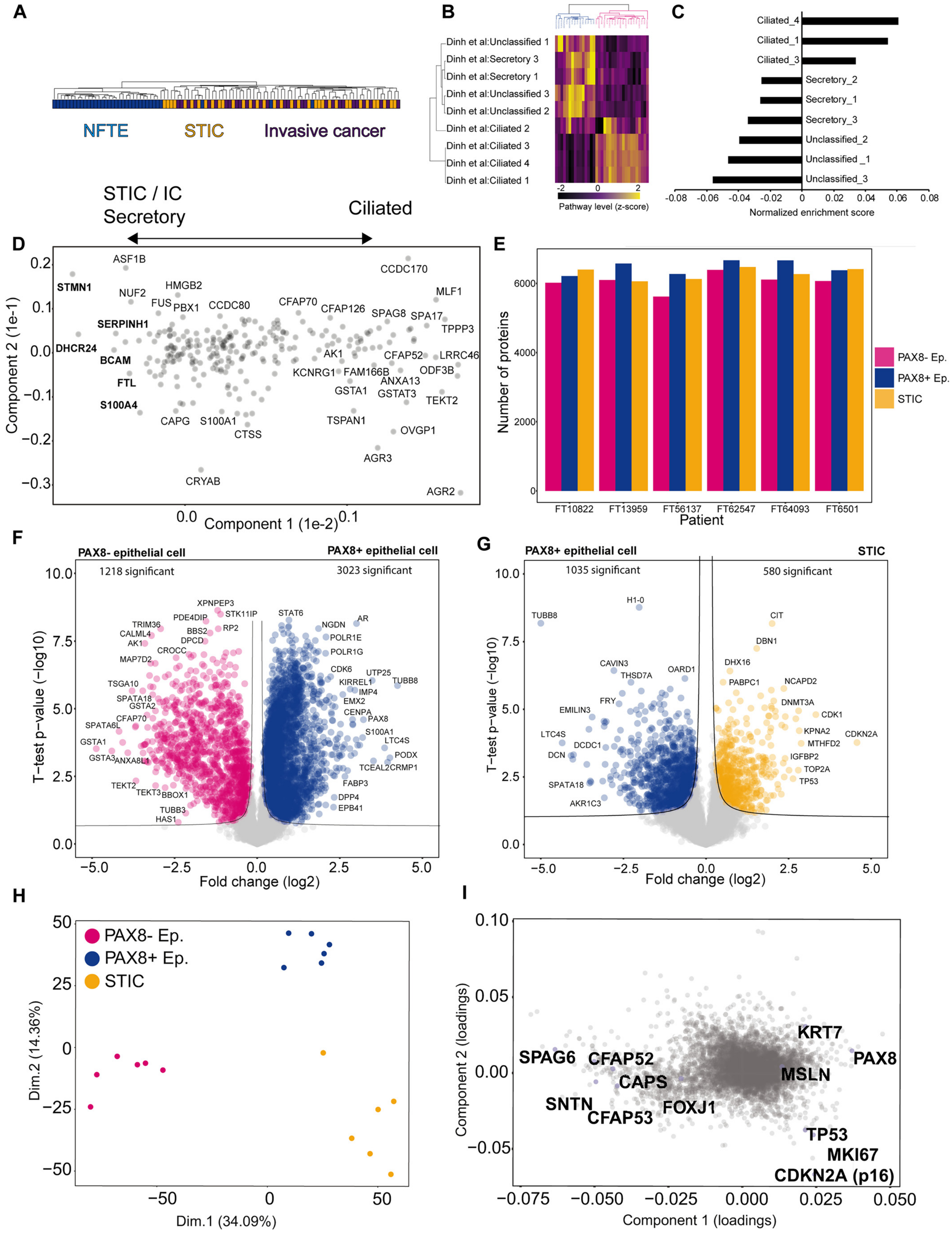
A
NFTE
STIC
Invasive cancer
B
Dinh et al:Unclassified 1
Dinh et al:Secretory 3
Dinh et al:Secretory 1
Dinh et al:Unclassified 3
Dinh et al:Unclassified 2
Dinh et al:Ciliated 2
Dinh et al:Ciliated 3
Dinh et al:Ciliated 4
Dinh et al:Ciliated 1
Pathway level (z-score)
C
Ciliated_4
Ciliated_1
Ciliated_3
Secretory_2
Secretory_1
Secretory_3
Unclassified_2
Unclassified _1
Unclassified_3
Normalized enrichment score
STIC / IC
Secretory
Ciliated
D
Component 2 (1e-1)
Component 1 (1e-2)
E
Number of proteins
Patient
PAX8- Ep.
PAX8+ Ep.
STIC
F
PAX8- epithelial cell
PAX8+ epithelial cell
1218 significant
3023 significant
T−test p−value (−log10)
Fold change (log2)
G
PAX8+ epithelial cell
STIC
1035 significant
580 significant
H
PAX8- Ep.
PAX8+ Ep.
STIC
Dim.2 (14.36%)
Dim.1 (34.09%)
I
Component 2 (loadings)
Component 1 (loadings)

© The Author(s)

Figure EV3. Precursor lesions feature histological markers and cell-of-origin signatures.

(**A**) Hierarchical tree showing the clustering pattern across regions (normal fallopian tube epithelium, STIC, and invasive fallopian tube carcinoma), revealing three epithelial samples that are atypical lesions based on proteome profiling. (**B**) Unsupervised hierarchical clustering of all normal fallopian tube epithelial samples based on cell type abundance scores. Cell type abundance scores were calculated based on the annotation matrix algorithm (Perseus) for all fallopian tube epithelial cell types identified by Dinh et al Two main clusters of secretory and ciliated cell-enriched normal fallopian tube epithelium samples were identified. (**C**) Enriched cell type signatures along PC1, related to Fig. 3F. (**D**) PCA loadings related to Fig. 3F. Note that proteins on the left are higher in STIC, invasive carcinoma, and secretory-like epithelial cells, whereas proteins on the right are higher in ciliated epithelial cells. (**E**) Barplots showing the number of proteins identified in the PAX8 negative (dark pink), PAX8 positive (dark blue), and STIC (yellow) compartments across six patients. (**F**) Volcano plot of the pairwise proteomic comparison between PAX8 positive (dark blue, 6 samples) and PAX8 negative (dark pink, 6 samples) epithelial samples. Cell and functional markers with the highest abundance change and significance are highlighted (two-sided Student's *t*-test, FDR <0.05). (**G**) Volcano plot of pairwise proteomic comparison between STIC (yellow, 6 samples) and PAX8 positive (dark blue, six samples) samples. Cell and functional markers with the highest abundance change and significance are highlighted (two-sided Student's *t*-test, FDR <0.05). (**H**) PCA of all quantified proteins comparing STICs (yellow), secretory (dark blue), and ciliated cell (dark pink) samples. (**I**) PCA loadings show known secretory and ciliated markers. Related to panel (**H**).

 © The Author(s)

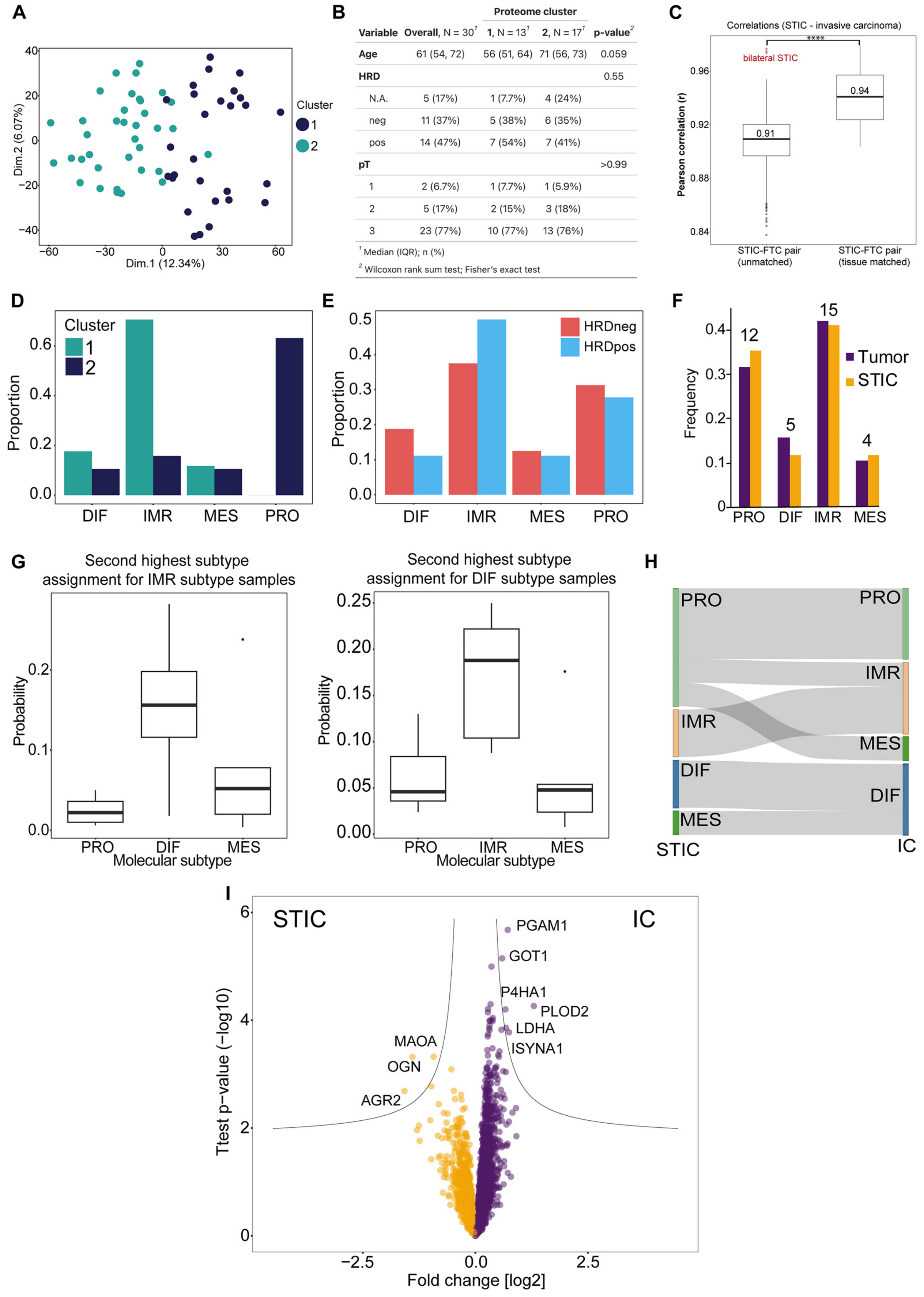

Variable	Overall, N = 30[1]	Proteome cluster 1, N = 13[1]	Proteome cluster 2, N = 17[1]	p-value[2]
Age	61 (54, 72)	56 (51, 64)	71 (56, 73)	0.059
HRD				0.55
N.A.	5 (17%)	1 (7.7%)	4 (24%)	
neg	11 (37%)	5 (38%)	6 (35%)	
pos	14 (47%)	7 (54%)	7 (41%)	
pT				>0.99
1	2 (6.7%)	1 (7.7%)	1 (5.9%)	
2	5 (17%)	2 (15%)	3 (18%)	
3	23 (77%)	10 (77%)	13 (76%)	

[1] Median (IQR); n (%)

[2] Wilcoxon rank sum test; Fisher's exact test

© The Author(s)

Figure EV4. Refined molecular subtyping from spatial proteomics data.

(**A**) Principal component analysis of STIC and invasive carcinoma samples corresponding to Fig. 3A. (**B**) Multivariate statistical analysis of patients in clusters 1 and 2. Note that cluster 2 samples showed a trend towards higher patient age at diagnosis ($p = 0.059$). (**C**) Boxplots showing proteomic correlations between STIC and invasive carcinoma samples between matched and unmatched samples, corresponding to intrapatient and interpatient correlations. Boxplots define the range of the data (whiskers), 25th and 75th percentiles (box), and medians (solid line). (**D**) Molecular subtype assignment of cluster 1 and 2 samples based on the consensusOV algorithm(Chen et al, 2018). Only samples with a margin score of >0.2 were included for high classification confidence. (**E**) Molecular subtype assignment of HRD-positive and HRD-negative invasive cancer samples based on the consensusOV algorithm(Chen et al, 2018). (**F**) Frequency of molecular subtypes of STIC and IC proteomes. (**G**) Boxplots indicating the second-highest molecular subtype classification probabilities for samples with immunoreactive (left) and differentiated (right) subtype calls. Boxplots define the range of the data (whiskers), 25th and 75th percentiles (box), and medians (solid line). Number of samples per molecular subtype with margin score >0.2: DIF – 5, PRO – 8, IMR – 5, MES – 2. (**H**) Sankey plot showing convergence or divergence of molecular subtypes for patient-matched STIC and invasive carcinoma pairs. (**I**) Volcano plot of pairwise proteomic comparison between STIC (yellow) and invasive fallopian tube carcinoma (violet) samples. Markers with the highest abundance change and significance are highlighted (two-sided Student's *t*-test, FDR <0.05). Number of samples per group: STIC – 35, IC – 31.

© The Author(s)

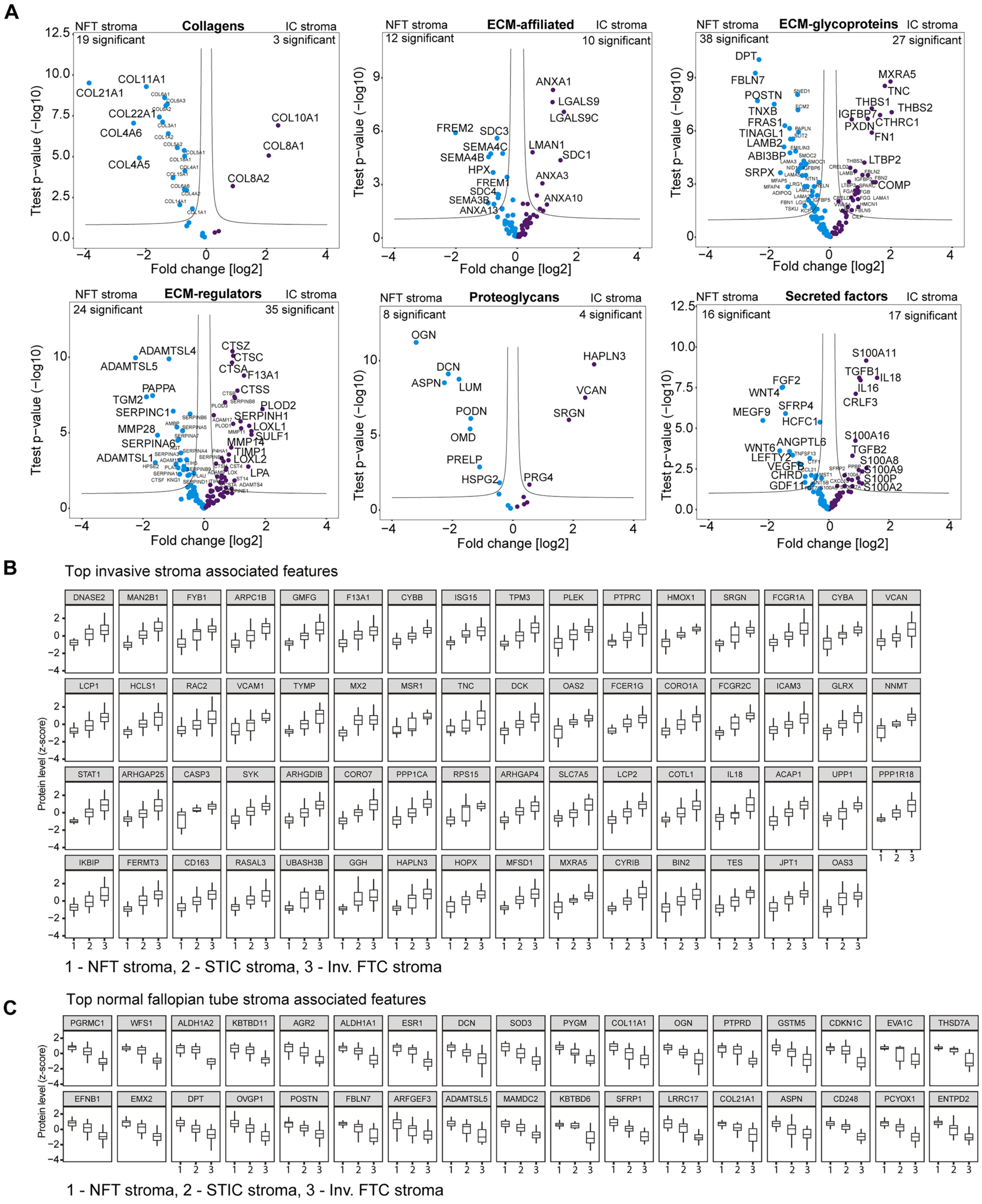
A
Collagens
NFT stroma
19 significant
IC stroma
3 significant
COL11A1
COL21A1
COL22A1
COL4A6
COL4A5
COL10A1
COL8A1
COL8A2
ECM-affiliated
12 significant
10 significant
ANXA1
LGALS9
LGALS9C
FREM2
SDC3
SEMA4C
SEMA4B
LMAN1
SDC1
HPX
ANXA3
FREM1
SDC4
SEMA3B
ANXA13
ANXA10
ECM-glycoproteins
38 significant
27 significant
DPT
FBLN7
POSTN
TNXB
FRAS1
TINAGL1
LAMB2
ABI3BP
SRPX
MXRA5
TNC
THBS1
IGFBP7
THBS2
PXDN
CTHRC1
FN1
LTBP2
COMP
ECM-regulators
24 significant
35 significant
ADAMTSL4
ADAMTSL5
CTSZ
CTSC
CTSA
F13A1
PAPPA
TGM2
CTSS
SERPINC1
PLOD2
SERPINH1
MMP28
LOXL1
SULF1
SERPINA6
MMP14
TIMP1
ADAMTSL1
LOXL2
LPA
Proteoglycans
8 significant
4 significant
OGN
DCN
ASPN
LUM
HAPLN3
VCAN
PODN
SRGN
OMD
PRELP
HSPG2
PRG4
Secreted factors
16 significant
17 significant
S100A11
TGFB1
IL18
FGF2
WNT4
IL16
CRLF3
MEGF9
SFRP4
HCFC1
S100A16
WNT6
ANGPTL6
TGFB2
LEFTY2
S100A8
VEGFB
S100A9
CHRD
S100P
GDF11
S100A2
Ttest p-value (−log10)
Fold change [log2]
B
Top invasive stroma associated features
DNASE2 MAN2B1 FYB1 ARPC1B GMFG F13A1 CYBB ISG15 TPM3 PLEK PTPRC HMOX1 SRGN FCGR1A CYBA VCAN
LCP1 HCLS1 RAC2 VCAM1 TYMP MX2 MSR1 TNC DCK OAS2 FCER1G CORO1A FCGR2C ICAM3 GLRX NNMT
STAT1 ARHGAP25 CASP3 SYK ARHGDIB CORO7 PPP1CA RPS15 ARHGAP4 SLC7A5 LCP2 COTL1 IL18 ACAP1 UPP1 PPP1R18
IKBIP FERMT3 CD163 RASAL3 UBASH3B GGH HAPLN3 HOPX MFSD1 MXRA5 CYRIB BIN2 TES JPT1 OAS3
Protein level (z-score)
1 - NFT stroma, 2 - STIC stroma, 3 - Inv. FTC stroma
C
Top normal fallopian tube stroma associated features
PGRMC1 WFS1 ALDH1A2 KBTBD11 AGR2 ALDH1A1 ESR1 DCN SOD3 PYGM COL11A1 OGN PTPRD GSTM5 CDKN1C EVA1C THSD7A
EFNB1 EMX2 DPT OVGP1 POSTN FBLN7 ARFGEF3 ADAMTSL5 MAMDC2 KBTBD6 SFRP1 LRRC17 COL21A1 ASPN CD248 PCYOX1 ENTPD2
Protein level (z-score)
1 - NFT stroma, 2 - STIC stroma, 3 - Inv. FTC stroma

◀ **Figure EV5. Mapping progressive ECM remodeling reveals stromal drug targets.**

(**A**) Volcano plot of pairwise proteomic comparison between IC stroma (purple, 29 samples) and NFT stroma (light blue, 31 samples) samples across different matrisome categories (collagens, ECM-affiliated, ECM glycoproteins, ECM regulators, proteoglycans, secreted factors). Markers with the highest abundance change and significance are highlighted (two-sided Student's *t*-test, FDR <0.05). (**B**, **C**) Boxplots of relative protein levels (group average, z-scored) for IC stroma (29 samples) (**B**) and NFT stroma (31 samples) (**C**)-associated features. Top-ranked protein features were identified by support vector machine classification, as shown in Fig. 5E. Boxplots define the range of the data (whiskers), 25th and 75th percentiles (box), and medians (solid line).

 © The Author(s)

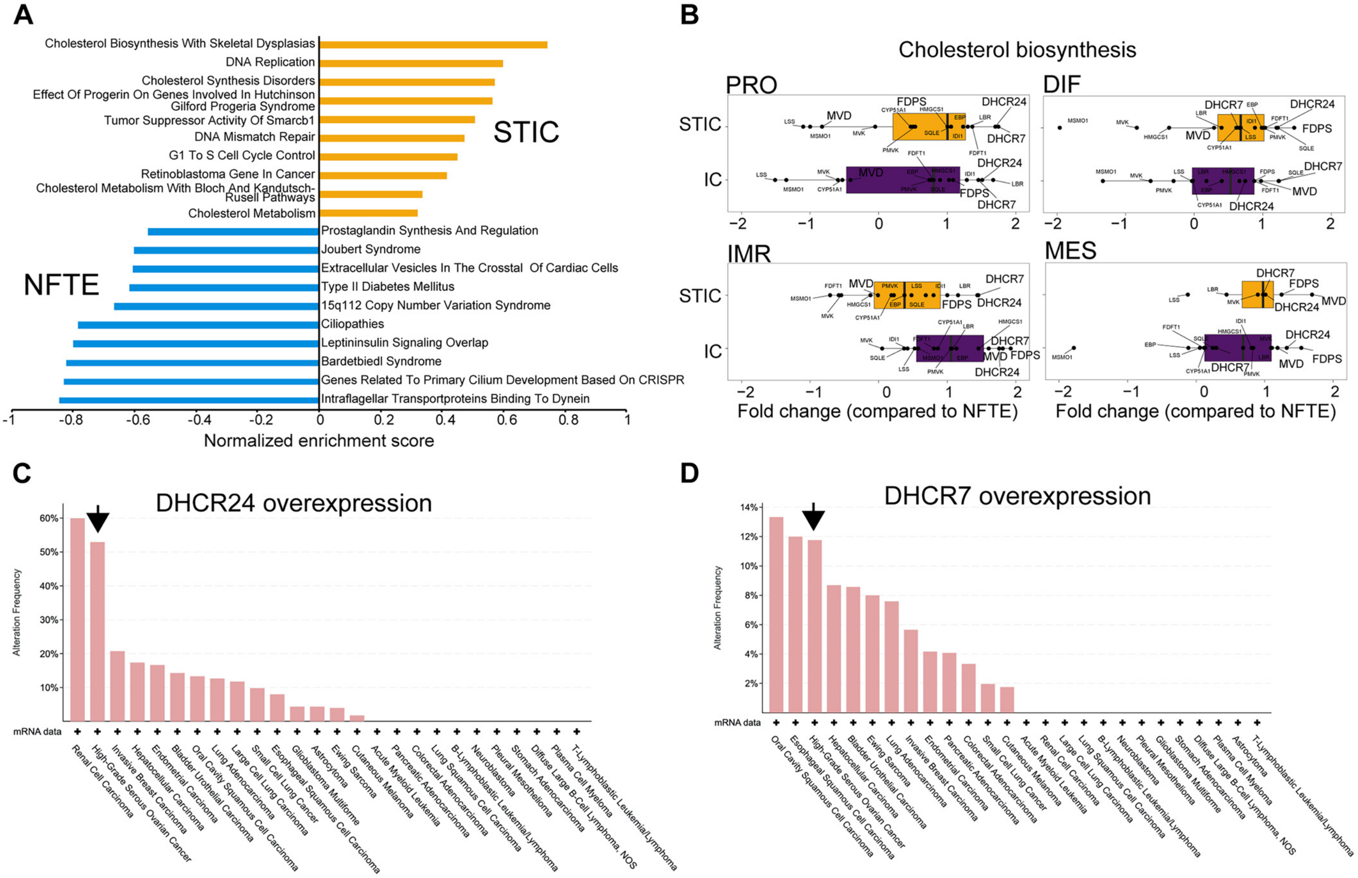

Figure EV6. Identification of commonly dysregulated pathways of therapeutic relevance.

(**A**) Pathway enrichment analysis (Hallmarks) based on t-test difference between STIC (yellow) and normal fallopian tube epithelium (light blue) samples. Selected pathways with a Benjamin–Hochberg FDR <0.05 are shown. (**B**) Protein fold changes (STIC vs. NFTE and IC vs NFTE, FDR <0.05) of all cholesterol biosynthesis pathway proteins across four molecular subtypes of HGSOC. Proteins with significant fold change are highlighted in bold. (**C**, **D**) Frequency of DHCR24 and DHCR7 genetic alteration (overexpression), respectively, across different cancer types analyzed by cBioPortal.

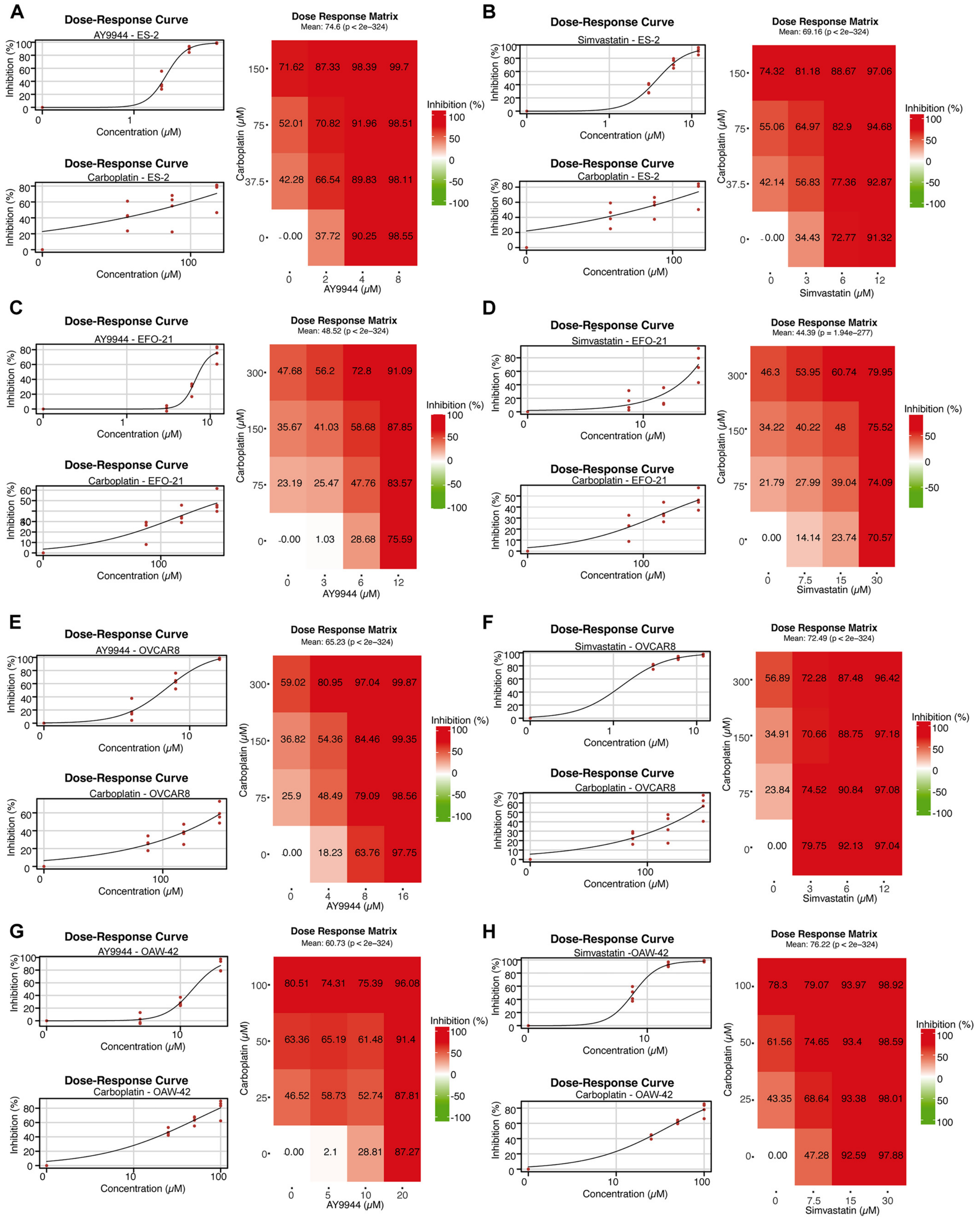
A
Dose-Response Curve
AY9944 - ES-2
Inhibition (%)
Concentration (μM)
Dose-Response Curve
Carboplatin - ES-2
Inhibition (%)
Concentration (μM)
Dose Response Matrix
Mean: 74.6 (p < 2e−324)
150 71.62 87.33 98.39 99.7
75 52.01 70.82 91.96 98.51
37.5 42.28 66.54 89.83 98.11
0 -0.00 37.72 90.25 98.55
0 2 4 8
AY9944 (μM)
Carboplatin (μM)
Inhibition (%)
100 50 0 -50 -100
B
Dose-Response Curve
Simvastatin - ES-2
Inhibition (%)
Concentration (μM)
Dose-Response Curve
Carboplatin - ES-2
Inhibition (%)
Concentration (μM)
Dose Response Matrix
Mean: 69.16 (p < 2e−324)
150 74.32 81.18 88.67 97.06
75 55.06 64.97 82.9 94.68
37.5 42.14 56.83 77.36 92.87
0 -0.00 34.43 72.77 91.32
0 3 6 12
Simvastatin (μM)
Carboplatin (μM)
Inhibition (%)
100 50 0 -50 -100
C
Dose-Response Curve
AY9944 - EFO-21
Inhibition (%)
Concentration (μM)
Dose-Response Curve
Carboplatin - EFO-21
Inhibition (%)
Concentration (μM)
Dose Response Matrix
Mean: 48.52 (p < 2e−324)
300 47.68 56.2 72.8 91.09
150 35.67 41.03 58.68 87.85
75 23.19 25.47 47.76 83.57
0 -0.00 1.03 28.68 75.59
0 3 6 12
AY9944 (μM)
Carboplatin (μM)
Inhibition (%)
100 50 0 -50 -100
D
Dose-Response Curve
Simvastatin - EFO-21
Inhibition (%)
Concentration (μM)
Dose-Response Curve
Carboplatin - EFO-21
Inhibition (%)
Concentration (μM)
Dose Response Matrix
Mean: 44.39 (p = 1.94e−277)
300 46.3 53.95 60.74 79.95
150 34.22 40.22 48 75.52
75 21.79 27.99 39.04 74.09
0 0.00 14.14 23.74 70.57
0 7.5 15 30
Simvastatin (μM)
Carboplatin (μM)
Inhibition (%)
50 0 -50
E
Dose-Response Curve
AY9944 - OVCAR8
Inhibition (%)
Concentration (μM)
Dose-Response Curve
Carboplatin - OVCAR8
Inhibition (%)
Concentration (μM)
Dose Response Matrix
Mean: 65.23 (p < 2e−324)
300 59.02 80.95 97.04 99.87
150 36.82 54.36 84.46 99.35
75 25.9 48.49 79.09 98.56
0 0.00 18.23 63.76 97.75
0 4 8 16
AY9944 (μM)
Carboplatin (μM)
Inhibition (%)
100 50 0 -50 -100
F
Dose-Response Curve
Simvastatin - OVCAR8
Inhibition (%)
Concentration (μM)
Dose-Response Curve
Carboplatin - OVCAR8
Inhibition (%)
Concentration (μM)
Dose Response Matrix
Mean: 72.49 (p < 2e−324)
300 56.89 72.28 87.48 96.42
150 34.91 70.66 88.75 97.18
75 23.84 74.52 90.84 97.08
0 0.00 79.75 92.13 97.04
0 3 6 12
Simvastatin (μM)
Carboplatin (μM)
Inhibition (%)
100 50 0 -50 -100
G
Dose-Response Curve
AY9944 - OAW-42
Inhibition (%)
Concentration (μM)
Dose-Response Curve
Carboplatin - OAW-42
Inhibition (%)
Concentration (μM)
Dose Response Matrix
Mean: 60.73 (p < 2e−324)
100 80.51 74.31 75.39 96.08
50 63.36 65.19 61.48 91.4
25 46.52 58.73 52.74 87.81
0 0.00 2.1 28.81 87.27
0 5 10 20
AY9944 (μM)
Carboplatin (μM)
Inhibition (%)
100 50 0 -50 -100
H
Dose-Response Curve
Simvastatin -OAW-42
Inhibition (%)
Concentration (μM)
Dose-Response Curve
Carboplatin - OAW-42
Inhibition (%)
Concentration (μM)
Dose Response Matrix
Mean: 76.22 (p < 2e−324)
100 78.3 79.07 93.97 98.92
50 61.56 74.65 93.4 98.59
25 43.35 68.64 93.38 98.01
0 0.00 47.28 92.59 97.88
0 7.5 15 30
Simvastatin (μM)
Carboplatin (μM)
Inhibition (%)
100 50 0 50 100

© The Author(s)

Figure EV7. Cholesterol biosynthesis inhibition sensitizes ovarian cancer cells to carboplatin.

(**A–H**) Dose–response curves (top left: AY9944 or simvastatin; bottom left: carboplatin) and dose–response matrices (right) for ES-2 (**A**, **B**), EFO-21 (**C**, **D**), OVCAR-8 (**E**, **F**), and OAW-42 (**G**, **H**) cells treated with AY9944 and carboplatin or simvastatin and carboplatin, respectively, alone or in combination. Experiments were performed in biological sextuplicate, each with $n = 3$ technical replicates.

© The Author(s)
